# Forgotten People, Forgotten Diseases: The Neglected Tropical Diseases and Their Impact on Global Health and Development

**DOI:** 10.3201/eid2810.220740

**Published:** 2022-10

**Authors:** Estee Y. Cramer, Andrew A. Lover

**Affiliations:** University of Massachusetts, Amherst, Massachusetts, USA

**Keywords:** bacteria, parasites, vector-borne infections, viruses, zoonoses, neglected tropical diseases, global health, media review, reference book

Forgotten People, Forgotten Diseases by Peter J. Hotez provides an overview of neglected tropical diseases (NTDs) that affect marginalized communities (Figure). NTDs are major global burdens but receive limited attention from researchers, policymakers, and funding agencies. Dr. Hotez is working to change this paradigm. He is a professor in the Departments of Pediatrics and Molecular Virology and Microbiology at Baylor College of Medicine, Houston, Texas; founding dean of the National School of Tropical Medicine at Baylor; and former president of the American Society for Tropical Medicine and Hygiene. Dr. Hotez shares his expertise in this clear and accessible text.

**Figure:**
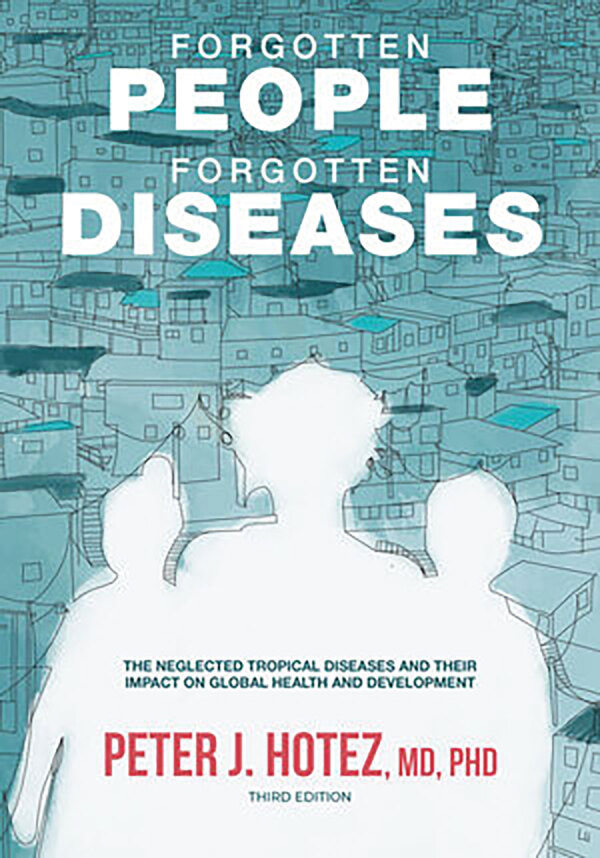
Forgotten People, Forgotten Diseases: The Neglected Tropical Diseases and Their Impact on Global Health and Development

This book begins by introducing the geographic distribution of NTDs and features that make these diseases stigmatizing, poverty-promoting, chronic, and often disabling. In subsequent chapters, Dr. Hotez addresses groups of related tropical diseases, such as soil transmitted helminths (Chapter 2) and mycobacterial infections (Chapter 6). Using vivid descriptions, he demonstrates vital reasons for treating these diseases, such as “each adult hookworm has the ability to fasten deeply to the inner lining of the intestine and extract blood… hookworms essentially rob growing children of their daily iron” (Chapter 2). Each chapter discusses morbidity and mortality (where known), essential features of biology and epidemiology, treatments, and public health control measures. Also included are current research, policy initiatives, economics of public health programs, and on-going global eradication efforts, including mass drug administration for lymphatic filariasis. He states, “In 2019 alone, more than 500 million people received [mass drug administration], representing almost two-thirds of the global population at risk” (Chapter 4).

The final chapters discuss current trends in NTD research and control. Although obstacles exist for validating and implementing treatments, Dr. Hotez praises pharmaceutical companies for donating albendazole and creating new facilities for NTD research (Chapter 11). This book concludes with a plea for tikkun olam, a Hebrew phrase for “repairing the world.” Dr. Hotez’s main objective is to inspire well-optimized interventions, such as including NTD control measures in US foreign policy, financial innovations, or government–academic–industrial enterprises devoted to NTDs (Chapter 12).

As a pioneer and global leader in NTD research and vaccine development, Dr. Hotez uses this book to highlight his unique perspective and research conducted with a large and diverse number of collaborators. As a clinician, he describes his experiences working with patients with NTDs. For example, he writes about patients who are concurrently affected by malnutrition and Chagas disease (Chapter 7).

The book could be improved by expanded discussion of the challenges to NTD elimination, such as the discovery of new paratenic hosts for dracunculiasis and ongoing effects of COVID-19. In addition, details provided for NTDs vary substantially. Almost an entire chapter is dedicated to schistosomiasis, but a single paragraph is dedicated to foodborne trematodes (Chapter 3). Additional information on the most neglected diseases would strengthen this text.

This book is useful for persons who are familiar with biology but have limited knowledge of NTDs, especially health economists, students, early-career global health researchers, and policymakers involved with NTD prevention, treatment, and elimination. Although the extent of NTDs is vast and diverse, Dr. Hotez conveys optimism throughout the text and projects a positive outlook toward ongoing research and elimination initiatives.

